# Case Report: Hypercholesterolemia “Lean Mass Hyper-Responder” Phenotype Presents in the Context of a Low Saturated Fat Carbohydrate-Restricted Diet

**DOI:** 10.3389/fendo.2022.830325

**Published:** 2022-04-14

**Authors:** Nicholas G. Norwitz, Adrian Soto-Mota, David Feldman, Stefanos Parpos, Matthew Budoff

**Affiliations:** ^1^ Harvard Medical School, Boston, MA, United States; ^2^ Metabolic Diseases Research Unit, National Institute for Medical Sciences and Nutrition Salvador Zubiran, Mexico City, Mexico; ^3^ Citizen Science Foundation, Las Vegas, NV, United States; ^4^ Elfers Cardiovascular Center, Mass-General Brigham Newton-Wellesley Hospital, Newton, MA, United States; ^5^ Department of Medicine, Tufts University School of Medicine, Boston, MA, United States; ^6^ Lundquist Institute at Harbor-UCLA Medical Center, Torrance, CA, United States

**Keywords:** carbohydrate restriction, coronary computed tomography angiography (CCTA), ketogenic diet, lean mass hyper-responder, LDL cholesterol, HDL cholesterol, triglycerides

## Abstract

Emerging evidence suggests that “leanness” and good metabolic health markers may predict larger increases in LDL cholesterol (LDL-C) in response to carbohydrate restriction. Specifically, a recent cohort study demonstrated an inverse association between BMI and LDL-C change among individuals on carbohydrate-restricted diets and identified a subgroup of “Lean Mass Hyper-Responders” (LMHR) who exhibit exceptional increases in LDL-C, in the context of low triglycerides and high HDL-C. We present the case of one subject, LM, who adopted a ketogenic diet for management of ulcerative colitis. He subsequently experienced an increase in LDL-C from 95 to 545 mg/dl, at peak, in association with HDL-C >100 mg/dl and triglycerides ~40 mg/dl, typical of the emergent LMHR phenotype. Assessments of LM’s dietary intake, lipid panels, and BMI are consistent with prior data and suggest that the LMHR phenomenon is not dependent on saturated fat intake but inversely associates with BMI changes. Finally, computed tomography angiography conducted on LM after over 2 years of hypercholesterolemia revealed no evidence of calcified or non-calcified plaque.

**Graphical Abstract:**
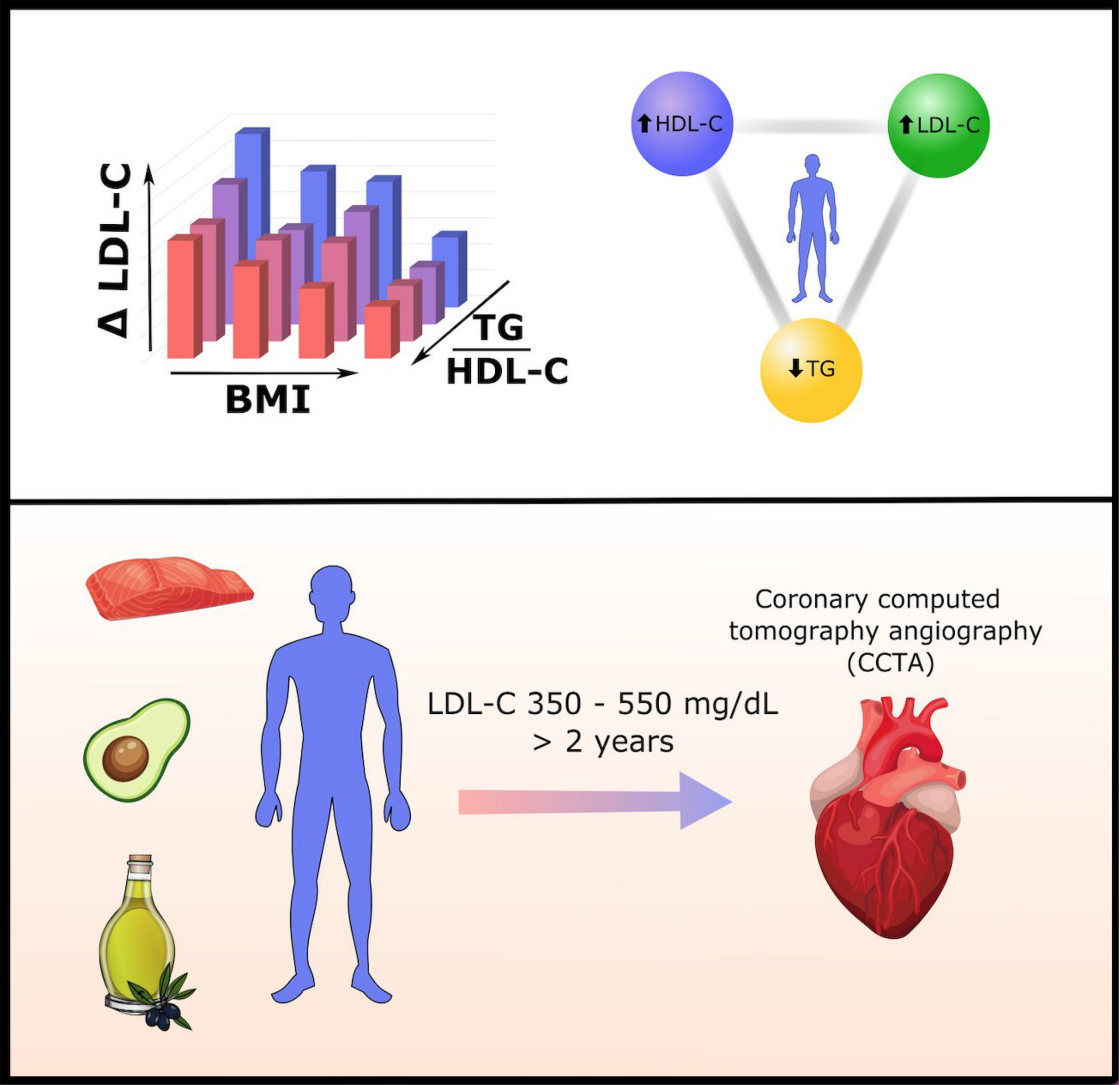
A recent cohort study of 548 persons on carbohydrate-restricted diets (CRDs) revealed inverse associations between triglyceride/HDL-C ratio (TG/HDL-C) and LDL-C change, and between BMI and LDL-C change, on CRD. This means leaner persons with lower TG/HDL-C ratios generally exhibit larger increases in LDL-C on CRD. Individuals with a particularly pronounced high LDL-C, high HDL-C, low TG ratio are termed "Lean Mass Hyper-Responders." This report provides a clinical vignette of a patient who exhibits the Lean Mass Hyper-Responder phenotype, with LDL-C as high as 545 mg/dl, despite normal pre-CRD LDL-C of 95 mg/dl and consuming a CRD with a high unsaturated/saturated fat ratio. Coronary CT angiography conducted after 2.5 years of extremely elevated LDL-C reveals no detectable plaque development.

## Introduction

In 2017, co-author Dave Feldman observed a trend: individuals who were lean and athletic tended to observe larger increases in LDL cholesterol (LDL-C) on carbohydrate-restricted diets (CRDs). He also noted that these cases of diet-induced increases in LDL-C co-occurred with increases in HDL-C and decreases in triglycerides (TG), giving rise to a combination lipid profile opposite to that of atherogenic dyslipidemia. The cut points chosen to characterize this phenotype included LDL-C ≥ 200, HDL-C ≥ 80, and TG ≤ 70 mg/dl, and were selected on the basis of empiric observation and based on the fact that each threshold is rare in the general population and, thus, the chance that any individual would pass all three cut points simultaneously—rather than as part of a triad—is highly unlikely. This lipid triad was termed the “Lean Mass Hyper-Responder” (LMHR) phenotype (1), given the apparent lean nature of individuals in whom it presented.

Since the proposal of the LMHR phenotype almost 5 years ago, an increasing number of people have been self-reporting to meet all three LMHR criteria. This trend may be, in part, driven by the increasing popularity of CRD and ketogenic diets for the management of non-obesity-related conditions, such as epilepsy, mood disorders, autoimmune and inflammatory bowel disease, as well as for general wellbeing or athletic performance. Indeed, there has been the emergence of an LMHR Facebook group of ~8,000 members and growing. However, whether the prevalence of this population is increasing, or we are simply now recognizing its existence, discussion of the phenotype has achieved medical community recognition as represented by reference to LMHR in prior case series by others (2, 3). However, until recently, the actual link between “leanness” and the aforementioned high LDL-C, high HDL-C, low TG lipid triad had not been systematically assessed.

We recently performed a hypothesis-naïve exploratory analysis on data drawn from a cohort of 548 people on CRD and demonstrated that there is an inverse relationship between BMI and changes in LDL-C (as well as between TG/HDL-C ratio and changes in LDL-C), in the context of carbohydrate restriction. Otherwise stated, leaner individuals with lower TG/HDL-C ratios are at greater risk for increases in LDL-C on a CRD (4). Notably, 18% (*n* = 100) of participants in this study fulfilled all three cut points of the LMHR phenotype, and were significantly leaner than other participants (Mean BMI, 22.0 ± 2.7 kg/m^2^ for LMHR vs. 24.6 ± 4.1 kg/m^2^ for non-LMHR, *p* = 1.2 × 10^−10^). These data were the first to formally characterize LMHR as a unique metabolic sub-group, paving the way for future study of LMHR individuals.

We herein present the case of a 26-year-old LMHR male, LM, who adopted a ketogenic diet prioritizing unsaturated fat for the management of ulcerative colitis and subsequently exhibited an increase in LDL-C of over 400 mg/dl, along with increases in HDL-C and decreases in TG. LM’s case possesses distinctive and useful features, including detailed information on dietary intake, longitudinal lipid panels, and a recent Coronary Computed Tomography Angiography (CCTA) scan, that collectively provide a valuable clinical vignette in the context of the recent classification of the LMHR phenotype. LM’s case also demonstrates the challenges associated with the clinical management of LMHRs and highlights the need for further study of those with this phenotype.

## Case Description

LM is a 26-year-old man with a medical history significant for ulcerative colitis, diagnosed at age 21. Despite treatment with oral and suppository mesalamine and hydrocortisone enemas, he was unable to remain in remission for longer than 8 weeks. At 23, he experimentally adopted a Mediterranean-style ketogenic diet [with an emphasis on intake of fatty seafood, extra virgin olive oil, and low-carbohydrate fibrous fruits and vegetables (5)] and entered a 6-month period of remission, following which he elected to discontinue all medications and has largely remained in remission. Since adopting a ketogenic diet two and a half years ago, LM has only experienced colitis flares on three occasions: twice when trying to reintroduce carbohydrates in the form of honey, fruit, and/or starchy vegetables, and once following acute mold exposure.

As previously reported (5), in the first 6 months following carbohydrate restriction, LM’s LDL-C increased from 95 mg/dl to 321 mg/dl, along with low TG and an increase in HDL-C from 48 to 109 mg/dl. This change occurred despite LM’s self-reported prioritization of foods rich in unsaturated fats, restricted intake of saturated fat-rich foods, such as red meat and dairy, and moderate fiber intake of ~30 g/day. Of note, LM subsequently reduced intake of fiber in order to manage constipation, and current intake is ~15 g/day.

When statin therapy was recommended to LM upon first presentation with hypercholesterolemia, he declined. While he expressed concern about his LDL-C levels, he also conveyed reluctance about consenting to potentially lifelong pharmacotherapy without first attempting to address his hypercholesterolemia with diet and lifestyle change. LM attempted on two occasions to reintroduce carbohydrates to lower his LDL-C. On both occasions, he experienced near-immediate gastrointestinal discomfort and blood in the stool within a week. Although he initially declined statins, he consented to trial ezetimibe but experienced gastrointestinal discomfort and discontinued treatment.

To further investigate potential contributing dietary factors to his hypercholesterolemia within the context of his ketogenic diet, in August 2020, LM began measuring his dietary intake using a gram food scale and by keeping fastidious dietary records in the 7 days prior to scheduled lipid testing. Nutritional information extracted from these records, using data exported from the USDA Food Central database along with brand-specific nutrition information, is provided with photographs of representative meals in **Supplemental Information**.

Lipid and metabolic panels over the years have been notable for elevated LDL-C-related markers, LDLp and apoB, and elevated Lp(a) ranging from 109 to 168 mg/dl. Despite his low LDL-C while consuming a mixed diet (95 mg/dl), Lp(a) was elevated in LM prior to starting a ketogenic diet and is also elevated in LM’s father, who has a history of coronary artery disease manifesting in 99% left anterior descending stenosis at age 44, although the father presented with a profile in strong contrast to his son: unremarkable LDL-C (70–92 mg/dl), atherogenic dyslipidemia (HDL-C 33–39 mg/dl, TG 199–294 mg/dl, with a preponderance of sdLDL), and history of obesity. (NB: Subsequent to his event, the father adopted a CRD and has lost 50 lbs and reversed metabolic syndrome, while maintaining LDL-C <100 mg/dl on atorvastatin and ezetimibe). LM exhibits normal thyroid and testosterone, HbA1c (4.8–5.0%), and fasting insulin (<3 μIU/ml), low hsCRP (<1.0 mg/L), Pattern A phenotype, and an extremely low TG/HDL-C ratio (~0.3) characteristic of the LMHR phenotype. Whole exome sequencing performed by Veritas Genetics, and independent dyslipidemia and ASCVD genetic risk testing by GB Healthwatch, revealed no pathogenic or likely pathogenic variants that could account for LM’s phenotype.

**Figure 1:**
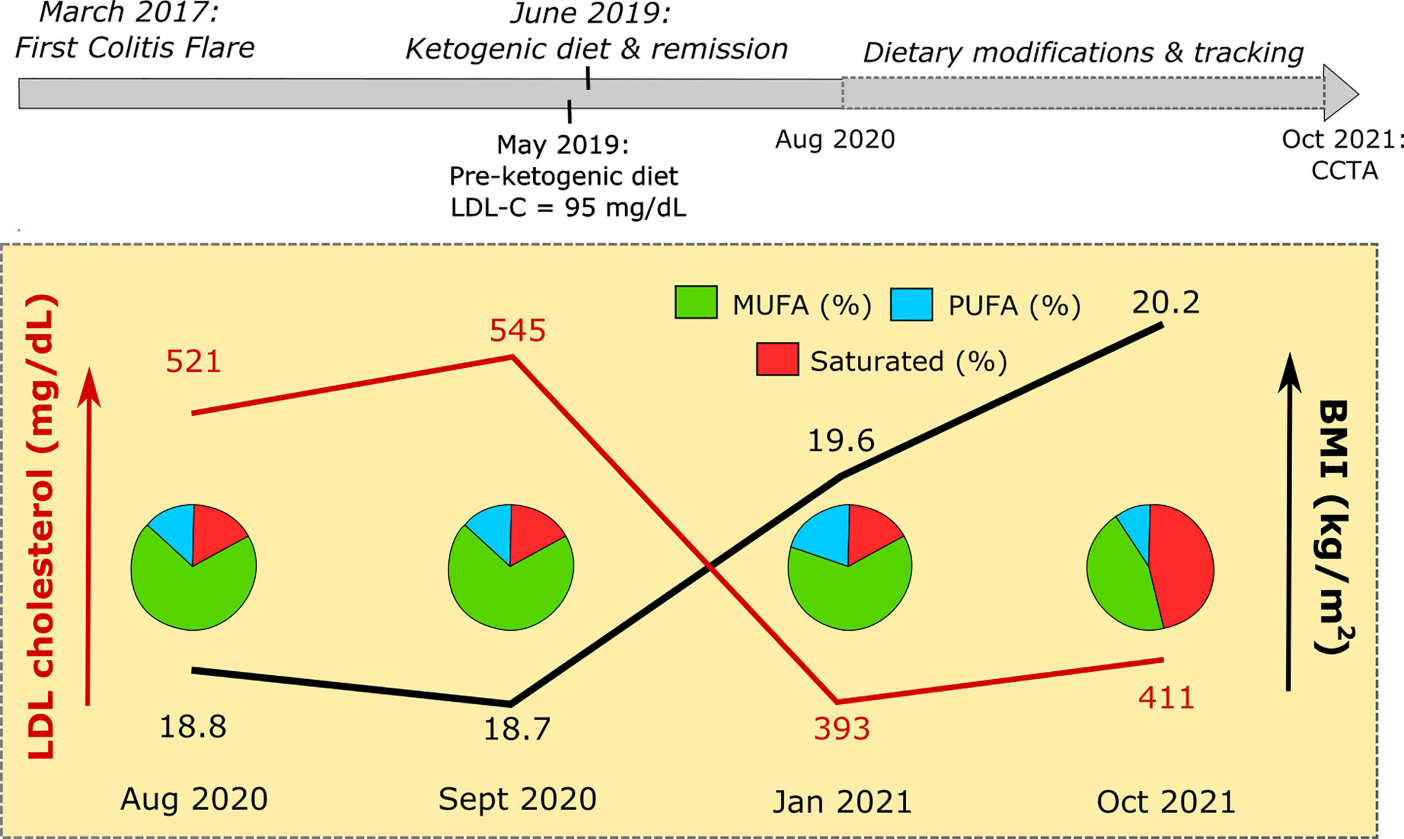
Patient timeline. Above: LM experienced his first colitis flare in March 2017. Episodic flares persisted, despite treatment with oral and suppository mesalamine and hydrocortisone enemas. LM commenced a ketogenic diet in June 2019, followed by a period of continued remission associated with ketosis. Medications for colitis were discontinued. Below: Between August 2020 and October 2021, LM began a practice of fastidiously recording dietary intake (see Supplemental Information) prior to scheduled lipid panels. Pie graphs represent fat pro!le of LM’s diet in the 7 days prior to testing. Further details provided in text, **Supplemental Information**, and **Table 1**.

Results of lipid panels conducted between August 2020 and October 2021, in conjunction with data extracted from LM’s dietary records, are presented in **Table 1** and **Figure 1**. In August 2020, LDL-C was 521 mg/dl (HDL-C 113 mg/dl, TG 47 mg/dl). This time point corresponds to LM’s BMI nadir of 18.8 kg/m^2^ and 83% unsaturated fat intake (17% saturated). In the following month, LM was recommended to reduce dietary cholesterol intake, eliminating liver, shellfish, and egg yolks from his diet (in substitution for lean chicken, fish, and egg whites). One month later, in September 2020, his LDL-C was re-measured at 545 mg/dl (HDL-C 94 mg/dl, TG 58 mg/dl). In January 2021, following a weight gain of 6 lbs and elevated intake of polyunsaturated fatty acid in the form of ~3 Tbsp toasted sesame oil daily, LDL-C was measured at 393 mg/dl (HDL-C 116 mg/dl, TG 40 mg/dl). Finally, testing in October 2021 following further weight gain of 4 lbs (BMI 20.2 kg/m^2^), and in the context of a diet far richer in saturated fat, LDL-C measured at 411 mg/dl (HDL-C 116 mg/dl, TG 39 mg/dl) (**Table 1** and **Figure 1**).

**Table 1:** Macronutrient intake and dietary fatty acid profile correspond to average daily intake over the 7 days prior to each blood test.

Date	TC	LDL-C(mg/dl)	HDL-C(mg/dl)	TG(mg/dl)	Weight (lb)	BMI (kg/m^2^)	Calories	Exercise (h)	Macronutrients (g)					Macronutrient (% Calories)				Dietary Fat Profile (%)		
									Net Carb	Protein	Fat	Fiber	Net Carb		Protein	Fat	Saturated		MUFA	PUFA
**May 2019 (Pre-keto)**	160	95	48	76	123	19.3	–	–	–	–	–	–	–		–	–	–		–	–
**Aug** **2020**	649	521	113	47	120	18.8	3,272	10.75	24	112	305	16	3		14	83	17		70	13
**Sept** **2020**	655	545	94	58	119	18.7	3,225	10.75	18	113	304	18	2		14	84	15		71	14
**Jan** **2021**	522	393	116	40	125	19.6	3,265	10.75	25	116	302	15	3		14	83	18		62	20
**Oct** **2021**	535	411	116	39	129	20.2	2,783	6.0	12	115	248	13	2		16	82	45		46	9

Calculations were performed using LM's dietary records (provided in **Supplemental Information**) and data extracted from USDA food central database in combination with brand-specific nutritional data, where applicable. Exercise volume is cumulative over the week and BMI was measured the morning of each test using the same Withings Smart Body Cardio scale. Lipid data prior to ketogenic diet are included for comparison, though dietary data were not collected prior to August 2020.

LM measured food, raw mass, using a kitchen gram scale.

**Figure 2:**
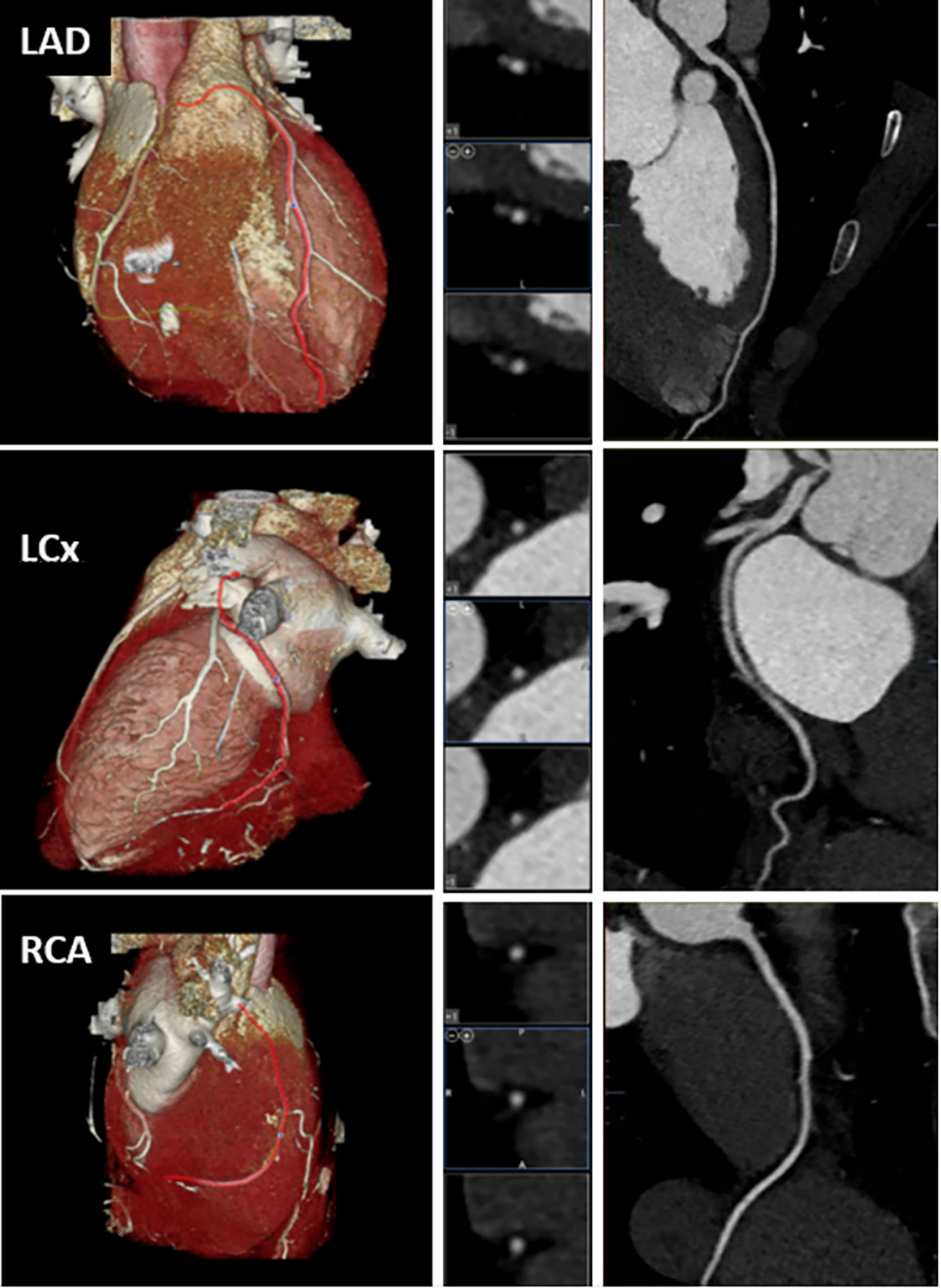
Computed tomographic angiogram demonstrating no coronary artery disease. Left column: Volume-rendered reformations. Middle column: Multiplanar reformation cross-sectional views. Right column: Curved multiplanar reformations. LAD, Left anterior descending artery; LCx, Left circumflex artery; RCA, Right coronary artery.

After two and a half years of persistently elevated LDL-C levels, and a prior CAC of 0, LM was again counseled to initiate statin therapy. He considered, and a compromise was reached whereby he agreed to initiate pharmacotherapy if “it was first proven” that he was developing measurable atherosclerotic plaque. Given consideration of data available at the time in young people at elevated risk for ASCVD (6), and LM’s significant exposure (LDL-C ~400–550 for ~2.5 years), a CCTA was ordered for calcified and non-calcified plaques. No plaque or stenosis was observed in any vessels CAD-RADS = 0 (**Figure 2**).

## Discussion

### In LM, LDL-C More Tightly Associated With Body Weight Than With Saturated Fat Intake

LM’s BMI and LDL-C data reveal that an increase of 6–10 lb body weight was associated with a >100 mg/dl drop in LDL-C. This presentation is consistent with prior cohort data (4), in which there was an inverse correlation between BMI and LDL-C among 548 people on CRD. However, as LM’s records are longitudinal, they provide the logical extension of our prior observation: they suggest that, for an LMHR on a CRD, becoming less lean could decrease LDL-C. The health implications of this potential strategy—weight gain—for lowering LDL-C in LMHR are unknown.

More notably, LM’s data demonstrate that the LMHR phenotype can exist in the context of a CRD that is relatively low in saturated fat. While this possibility was suggested by our cohort data (given the low likelihood that lean metabolically healthy participants selectively consumed CRDs richer in saturated fat, as compared with those with higher BMI and TG/HDL-C ratio on CRDs), we were previously unable to demonstrate that high relative intake of saturated fat is not required to produce the LMHR phenotype. However, LM’s dietary fat profiles were >80% unsaturated prior to the drawings of his two highest LDL-C (August and September 2020). Conversely, LM’s most recent labs, drawn following both weight gain and a marked increase saturated fat intake, reveal a relative decrease in LDL-C from peak. Thus, saturated fat intake is not a primary driver of LDL-C change in LM.

Although LM’s data cannot be said to be generalizable to all LMHR, they are consistent with prior data and suggest that BMI is a more important determinant of LDL-C in LMHR than is saturated fat intake.

### Lean Mass Hyper-Responders on Carbohydrate-Restricted Diets Constitute a Unique Subgroup of Individuals Not Represented in NHANES Population-Level Data

While it may be self-evident that the LMHR triad of LDL-C ≥ 200, HDL-C ≥ 80, and TG ≤ 70 mg/dl represents an atypical lipid profile, it is worth emphasizing that patients bearing this phenotype are extraordinarily rare among the general population. For example, our analysis of the 70,310 patients in the NHANES 1999–2020 database for which there are LDL-C, HDL-C, and TG data reveals 513 patients with LDL-C ≥ 200, 4,641 with HDL-C ≥ 80, and 19,345 with TG ≤ 70 mg/dl. However, only 3 cases passed the combined three criteria of the LMHR phenotype (the publicly available code for this analysis can be accessed at this link: https://github.com/AdrianSotoM/LMHR/blob/main/LMHRSinNHANEScode.R). By contrast, our study of 548 people consuming CRD revealed that 18% (*n* = 100 individuals) were LMHR (4). The juxtaposition of the rarity of LMHR throughout the general population (composed mostly of people who are overweight, metabolically unhealthy, and/or eating carbohydrate-rich diets) with the relative prevalence of LMHR in leaner people consuming CRD is consistent with the notion that the LMHR triad may reflect a unique underlying metabolic state.

### The Lipid Energy Model: An Explanation for the Lean Mass Hyper-Responder Phenomenon

If low BMI, or perhaps more accurately low relative adiposity, rather than elevated saturated fat intake drives the LMHR phenotype, the question that inevitably follows is “why?” The Lipid Energy Model is a hypothesis that could explain the LMHR phenomenon in patients like LM. While a formal description of the model is beyond the scope of this manuscript, we here offer a brief primer.

In the context of carbohydrate restriction, there is a greater demand by peripheral tissues for fat-based fuel. Aside from ketone bodies, this can come from one of two sources: (i) non-esterified fatty acids (NEFAs) released from adipocytes and (ii) TG-rich lipoproteins (TGRLs), including VLDL secreted by the liver. In the latter scenario, TGRLs undergo lipoprotein-lipase (LPL)-mediated hydrolysis to supply TG from the lipoprotein particles. The process of LPL-mediated remodeling of VLDL yields surface remnants and core remnants, which contribute to increases in HDL-C and LDL-C, respectively (7).

**Figure 3:**
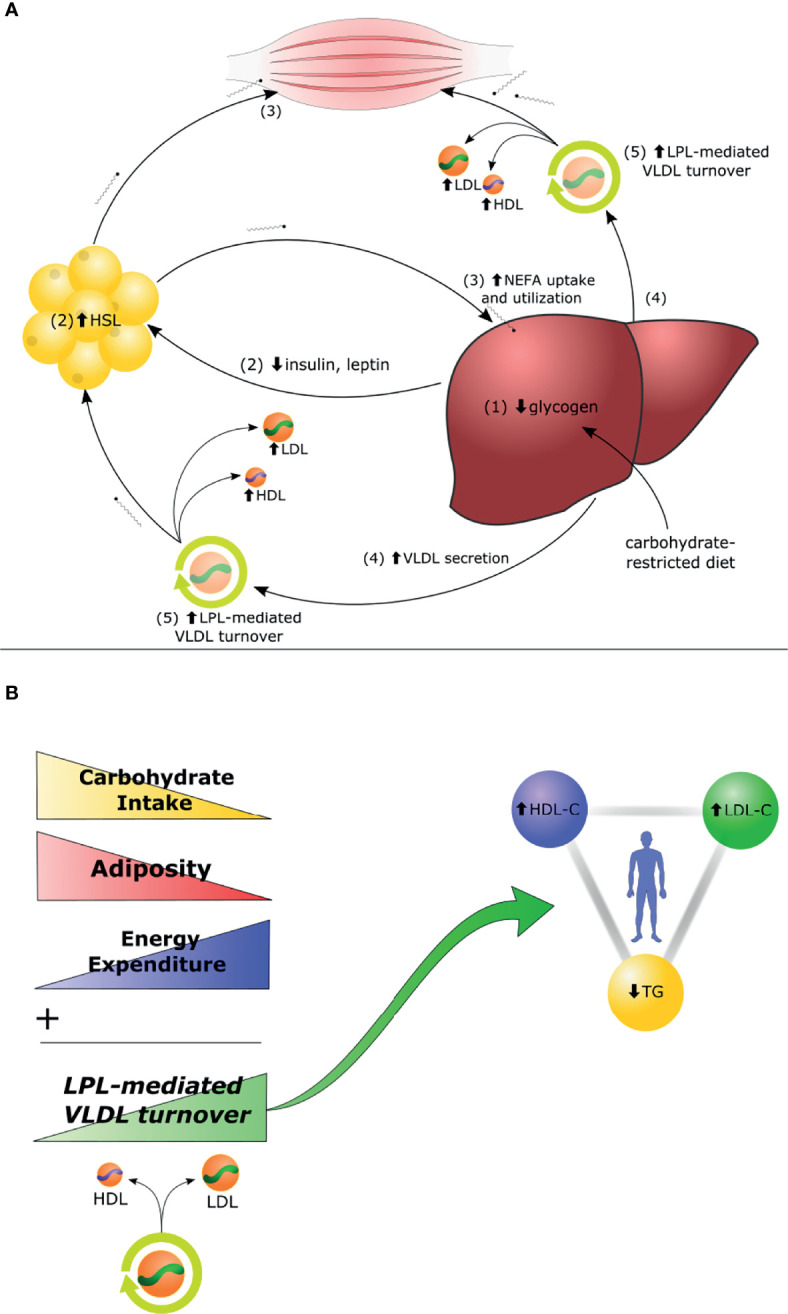
The Lipid Energy Model. **(A)** In the context of carbohydrate restriction, (1) glycogen depletion and (2) changes in circulating hormones stimulate hormone-sensitive lipase (HSL)-mediated secretion of non-esterified fatty acids (NEFA) by adipocytes to fuel oxidative tissues. (3) The liver captures circulating NEFA and repackages them into triglycerides (TG), (4) secreted aboard VLDL. (5) Increased lipoprotein-lipase (LPL)-mediated VLDL turnover generates increase of LDL-C and HDL-C. **(B)** The magnitude of carbohydrate restriction, adiposity, and energy expenditure each contribute as independent variables to the degree of LPL-mediated VLDL turnover and, thereby, to the magnitude of the high LDL-C, high HDL-C, low TG triad. Details and lines of evidence supporting this Lipid Energy Model, along with suggestions for future experiments to assess the model, will be the subject of an upcoming review (Norwitz et al. in preparation).

In brief, the Lipid Energy Model postulates that, in leaner metabolically healthy individuals, there is an increase in VLDL secretion, LPL-mediated TG hydrolysis and VLDL turnover, resulting in a profile of low TG, elevated HDL-C, and elevated LDL-C. Furthermore, the Lipid Energy Model predicts that the rate of LPL-mediated VLDL turnover is related to energy demands and inversely related to body fat mass, and thereby provides a useful framework for hypothesis generation and testing (**Figure 3**). Details and lines of evidence supporting this Lipid Energy Model will be the subject of an upcoming review (Norwitz et al. in preparation).

### Computed Tomography Angiography Shows No Evidence of Atherosclerotic Plaque in LM

LM’s CCTA data are difficult to interpret given his young age and the relative paucity of comparator data. At the time the scan was ordered (for the purposes of directing pharmacotherapy), it was reasonable to assume that he might exhibit plaque development based on the available data. This includes a study on young adults with type II diabetes, mean HbA1c 7.9%, in which 80% of those above 25 years exhibited measurable plaque burden (6), and, of course, that children with homozygous familial hypercholesterolemia and LDL-C levels comparable to LM can present with xanthoma and suffer from myocardial infarctions in the first decade of life (8). For a direct example, patient 2 in Luirink et al. (9) was diagnosed with homozygous familial hypercholesterolemia at age 0.9 years, with LDL-C of 548 mg/dl (similar to levels achieved by LM) and xanthomas (not present in LM). Following dual treatment with rosuvastatin and ezetimibe at 2.2 years and lipoprotein apheresis at 5.5 years, ultimately lowering LDL-C to 139 mg/dl, this patient’s first CCTA at age 8 years revealed RCA and LAD plaques. His years exposure to LDLC ~548 mg/dl (2.2 years) is similar to that of LM. Thus, without the benefit of hindsight, it was reasonable to assume LM, age 26, could exhibit plaque on CCTA. In retrospect, however, it can of course also reasonably be argued that, despite the magnitude of his exposure, two and half years is insufficient for any measurable atherosclerotic plaque to precipitate. The absence of comparator data itself highlights the need for further study on the LMHR population.

## Limitations and Strengths

LM’s case is subject to all the interpretive limitations of any case report. As singular patient data, his results cannot be assumed to be generalizable and are subject to the potential influences of individual genetics, diets, medication, disease stage, confounding lifestyle factors, and so on. These data should also not be taken out of context and analogized to conditions of high LDL-C/apoB in people consuming mixed diets. Furthermore, LM’s age obscures interpretation of his negative CCTA and it is possible that he is at significantly increased risk of ASCVD relative to other young adults. Nevertheless, LM’s case is notable for that patient’s fastidious records, which suggest that the LMHR phenotype can exist in the context of a CRD low in saturated fat, and for his negative CCTA data, which are difficult to interpret but highlight the value of an upcoming double-blinded prospective study that is already underway to assess atherosclerotic plaque progression in a sample of 100 LMHR.

## Conclusion

As suggested by our recent cohort data, the LMHR phenotype is unique in that their high LDL-C appears (i) to be dependent on dietary fuel preference, (ii) to be independent of known genetic factors, (iii) to be inversely related to BMI, and (iv) to occur particularly in the context of low TG/HDL-C ratio. The phenotype is also becoming increasingly common and more widely recognized in conjunction with the increasing popularity of CRD and ketogenic diets. The case of LM is consistent with features of the phenotype as noted above (including independence from saturated fat intake in this individual) and further provides a clinical vignette emphasizing the mechanistic curiosities and clinical complexities associated with LMHR phenotype.

## Patient Perspective

When I started my ketogenic diet, I regained a quality of life that I thought I had lost forever. Over the two prior years, my colitis symptoms were getting steadily worse, and I was losing the ability to function, both academically and socially. Imagine a life in which you had to continuously worry about having an embarrassing and painful bathroom emergency at any time: during an exam, or a car trip, on a date. How would you feel? My ketogenic lifestyle did not just erase my physical symptoms, it gave me back a life.

The rise in my LDL-C was an unforeseen consequence. At the time, I had never heard of “Lean Mass Hyper-Responders”, and did not believe LDL-C could increase as high as mine did as a result of diet. To my surprise, this phenomenon is somewhat common and, as it turns out, there are thousands of people like me who are lean individuals who adopted a ketogenic diet and found that they felt better than they had in years, but at the “cost” of exorbitantly high LDL-C.

As someone who considers himself scientifically inclined, I find the LMHR phenotype both scary and fascinating. This appears to be a reproducible phenomenon in which lean people specifically are able to manipulate their lipid levels to an astonishing degree by shifting the macronutrient composition of their diet. What is the mechanism and what are the implications?

I have also noticed that for many others who share my situation, this is neither something we have actively chosen nor is it an issue most of us take lightly. Like myself, many have sought other means of reducing LDL-C but have conditions making it uniquely challenging for them. Speaking for myself, I only want answers as to why my LDL-C increased and, more importantly, greater insight into the risk associated with my LDL-C levels in myself as an individual patient. One can certainly and fairly argue the preponderance of evidence supports lowering LDL-C, but I would also argue that LMHR are a unique subgroup that at least deserves further study so that patients like me can make more informed decisions, rather than feeling like we are getting brushed under the rug.

## Data Availability Statement

The original contributions presented in the study are included in the article/**Supplementary Material**. Further inquiries can be directed to the corresponding author.

## Ethics Statement

Ethical review and approval were not required for the study on human participants in accordance with the local legislation and institutional requirements. The patients/participants provided their written informed consent to participate in this study. Written informed consent was obtained from the individual(s) for the publication of any potentially identifiable images or data included in this article.

## Author Contributions

All authors listed have made a substantial, direct, and intellectual contribution to the work and approved it for publication.

## Conflict of Interest

NN is coauthor of a Mediterranean low-carbohydrate diet cookbook; he donates all royalty payments to nutrition research and education. DF is a partner in Own Your Labs LLC but is not on the payroll and contributes all proceeds to the Citizen Science Foundation. MB has grant support from General Electric.

The remaining authors declare that the research was conducted in the absence of any commercial or financial relationships that could be construed as a potential conflict of interest.

## Publisher’s Note

All claims expressed in this article are solely those of the authors and do not necessarily represent those of their affiliated organizations, or those of the publisher, the editors and the reviewers. Any product that may be evaluated in this article, or claim that may be made by its manufacturer, is not guaranteed or endorsed by the publisher.

## Abbreviations

CCTA, Coronary Computed Tomography Angiography; CRD, Carbohydrate-restricted diet; HDL-C, HDL cholesterol; LDL-C, LDL cholesterol; LMHR, Lean Mass Hyper-Responder; NEFA, Non-esterified fatty acids; TG, Triglycerides; TGRL, TG-rich lipoproteins; VLDL, Very-low-density lipoprotein.
