# Supplementary material for: Case Report: Hypercholesterolemia “Lean Mass Hyper-Responder” Phenotype Presents in the Context of a Low Saturated Fat Carbohydrate-Restricted Diet
Source: Front Endocrinol (Lausanne). 2022 Apr 14;13:830325. doi: 10.3389/fendo.2022.830325 (PMC9048595; doi:10.3389/fendo.2022.830325)
Supplement: Supplementary file 1 [file DataSheet_1.docx]

# Supplemental Information

**Patient dietary records**. Daily intakes are recorded in sheets, “Day1-7,” for the seven days prior to lipid testing. The patient attests to measuring food in raw weight using a gram scale. Nutritional information is drawn from sheet “My_Library,” with per entry item data drawn from USDA food


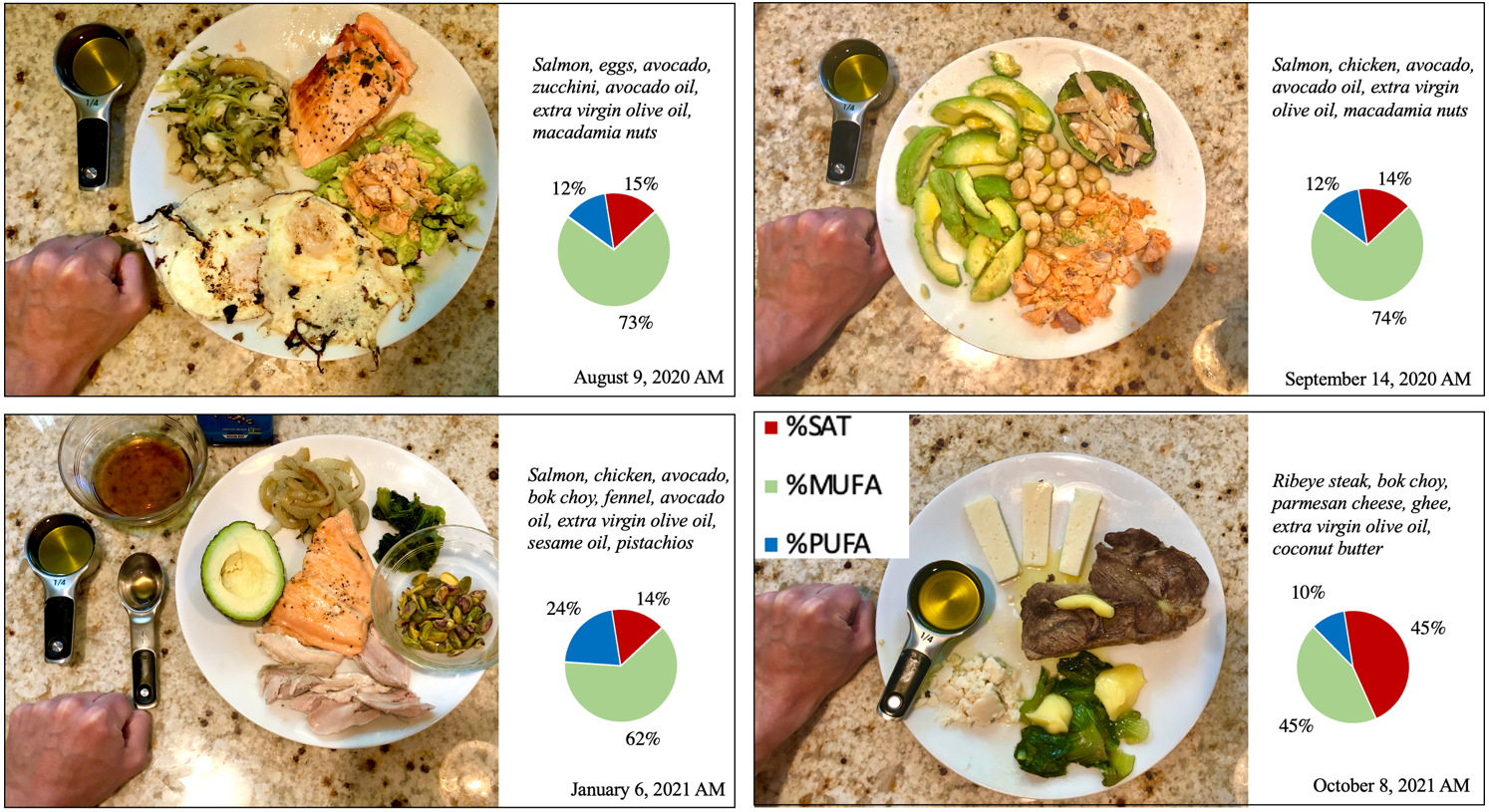


**Representative meal images**. Photographs of the patient’s four representative breakfast meals, taken on the sixth day of each week prior to lipid testing. Pie charts show the fatty acid profile of each meal. Additional nutritional details are provided in the supplemental dietary records.
